# Sparse Component Analysis Using Time-Frequency Representations for Operational Modal Analysis

**DOI:** 10.3390/s150306497

**Published:** 2015-03-17

**Authors:** Shaoqian Qin, Jie Guo, Changan Zhu

**Affiliations:** Department of Precision Machinery and Precision Instrumentation, University of Science and Technology of China (USTC), Hefei 230027, China;E-Mails: qinshaoq@mail.ustc.edu.cn (S.Q.); changan@ustc.edu.cn (C.Z.)

**Keywords:** sparse component analysis, blind source separation, operational modal analysis, single source points

## Abstract

Sparse component analysis (SCA) has been widely used for blind source separation(BSS) for many years. Recently, SCA has been applied to operational modal analysis (OMA), which is also known as output-only modal identification. This paper considers the sparsity of sources' time-frequency (TF) representation and proposes a new TF-domain SCA under the OMA framework. First, the measurements from the sensors are transformed to the TF domain to get a sparse representation. Then, single-source-points (SSPs) are detected to better reveal the hyperlines which correspond to the columns of the mixing matrix. The K-hyperline clustering algorithm is used to identify the direction vectors of the hyperlines and then the mixing matrix is calculated. Finally, basis pursuit de-noising technique is used to recover the modal responses, from which the modal parameters are computed. The proposed method is valid even if the number of active modes exceed the number of sensors. Numerical simulation and experimental verification demonstrate the good performance of the proposed method.

## 1. Introduction

During the last few decades, modal analysis has been a useful analytical tool in linear dynamical systems. Modal information, including natural frequency, damping ratio and mode shape, can be extracted through modal identification to completely characterize the dynamics of a linear system. Output-only modal identification or operational modal analysis (OMA) has attracted the interest of many researchers in structural dynamics because of its capacity to extract modal information from output measurements alone [1–3].

Modal identification coincides with the concept of blind source separation(BSS), which is the process of extracting original sources and mixing matrix from output signals without prior knowledge of the mixing procedures or sources. The linear instantaneous-mixing BSS model can be formulated as

(1) x(t)=As(t)

where **x**(*t*) is a column vector of *m*, zero mean, and output observations that represent an instantaneous linear mixture of source signals in column vector **s**(*t*); **A** ∈ ℝ*^m^*^×^*^n^* is an *m* × *n* matrix, which is referred to as the mixing matrix. The observation vector x(*t*) may be construed as a superposition of the columns of matrix **A** loaded by individual sources **s***_i_*(*t*), *i* = 1, … , *n*. The objectivity of BSS is to recover sources **s**(t) from observations **x**(*t*) only without any knowledge of **A**. From the perspective of modal expansion, the BSS problem has a similar form to OMA, in which the output responses are a linear combination of decoupling monotone modes viewed as source signals.

Many studies have attempted to use BSS for OMA [4–8]. [4] was the first to recognize the correlation between the vibration modes of mechanical systems and the sources computed through independent component analysis(ICA); ICA is effective in weakly damped modes(with less than 1% damping). Classical second-order BSS(SOBSS) algorithms such as multiple unknown signal extraction(AMUSE) and second-order blind identification(SOBI) were successfully applied to modal identification and enhanced damped mode separation [5,6]. [7,9] highlighted the limitations of SOBI and proposed a framework using Hilbert transform to estimate complex mode shapes. [10] proposed a method that combines SOBI and state-space realization on the basis of the non-Hermitian joint approximate diagonalization of the correlation matrices at several time lags; the method established a theoretical link between SOBSS and stochastic subspace identification(SSI) [2,11], which is a popular modal identification algorithm.

Unlike traditional methods, BSS is straightforward, computationally efficient and can function unsupervised; however, it can estimate only as many active modes as the number of output responses [12]. The BSS problem that occurs when the number of sensors is less than the number of the sources is referred to as underdetermined BSS(UBSS), which has received much attention in recent years [10,13]. Sparse component analysis(SCA) [14–16] was recommended to solve UBSS. Unlike traditional BSS methods that rely on source independence, SCA requires sources to have sparsity in the time domain or some transformed domain. Many studies proposed algorithms based on the sparsity in the TF domain [17–22]. In [17], singular value decomposition was used on wavelet coefficients to identify frequencies and damping ratios; this method is faster than other ridge detection algorithms. In [19], principal component analysis was performed on the wavelet coefficients to produce the partial mixing matrix of UBSS.

Speech signals are highly transient, consisting of voiced segments exhibiting a well-defined frequency structure, which are separated by noise bursts. In each short period of time, only a small number of frequency bands hold most of the energy, while other frequency bands contain little energy. Therefore, if the speech signals are transformed to time-frequency domain, magnitude of most of the time-frequency coefficients in the time window are approximate to zero, which meets the sparsity condition of SCA; hence SCA has been widely studied in speech processing [23–30]. [23,24] propsed the degenerate types of the unmixing estimation techniques under the assumption that the W-disjoint orthogonal condition occurs across the whole TF region. [25,26] proposed the types of time-frequency ratio of mixtures(TIFROM) methods to relax the restrictive orthogonal assumption. The TF coefficients of sources were allowed to overlap, but adjacent TF regions where only one source occurs must exist. In [27], the restriction of the TIFROM algorithm was further relaxed because only two adjacent points with a single source contribution in the same frequency bin were demanded. This relaxation was based on the fact that the magnitude of the mixture vector at each single-source-point(SSP) is proportional to that of one of the mixing column vectors. [28] reported a simple algorithm of identifying SSPs through hierarchical clustering. This algorithm achieved high estimation performance which could be further improved by using highly suitable clustering algorithms.

In OMA, the sources are exponentially-decaying sinusoids with different instinct frequencies, the sparse property of the sources in frequency or TF domain makes it suitable to execute modal identification using SCA. [31] studied SCA by using the sparsity of modal responses in the frequency domain, and estimated the mixing matrix and modal parameters. Instead of exploring frequency-domain sparsity, the present study investigates sparse representations in the TF domain. The proposed SCA consists of two steps: estimation of the mixing matrix and reconstruction of sources. The mixed signals are first transformed into the TF domain. The scatter plot of the TF coefficients reveals the hyperlines that correspond to the column vectors of the mixing matrix because of sparsity. The mixing matrix can be estimated through cluster analysis. SSP techniques [28] and K-hyperline clustering are combined to improve the performance of SCA because the estimation of the mixing matrix plays a key role in the latter reconstruction of sources. Finally, the theory of linear equations for determined BSS or basis pursuit de-noising (BPDN) for UBSS is used to recover the modal responses. Modal parameters are then estimated by using the single-degree-of-freedom(SDOF) parameter fitting method. The main contribution of this paper is to introduce the TF-SSP technique to OMA. The procedure of extracting SSPs can eliminate the points which don't lie in hyperlines and make estimation of mixing matrix more accurate, especially in the presence of noise. Numerical and experimental results show the accurate and robust identification performance of the method.

The paper is structured as follows. Section 2 introduces the theory of modal expansion, and Section 3 presents the derivation of SCA. Sections 4 and 5 provide the numerical simulations and experimental verification, respectively. The conclusion is drawn in Section 6.

## 2. Modal Expansion and BSS

For an n-degree-of-freedom linear system, the governing equations can be written as

(2) $$M\ddot{\text{x}}\left(t\right)+C\dot{\text{x}}\left(t\right)+K\text{x}\left(t\right)=f\left(t\right)$$

where **M** ∈ ℝ *^n^*^×^*^n^* is the mass matrix, **C** ∈ ℝ *^n^*^×^*^n^* is the damping matrix, and **K** ∈ ℝ *^n^*^×^*^n^* is the stiffness matrices; **f**(*t*) is the external force, **x** is the *n*-dimensional displacement response vector, and dot denotes derivatives with respect to time.

On the basis of modal expansion theory, the system responses **x**(*t*) = [*x*_1_(*t*),… ,*x_n_*(*t*)]*^T^* with diagonalizable damping that can be expressed as

(3) $$\text{x}\left(t\right)=\Phi q\left(t\right)=\sum_{i=1}^{n}\varphi_{i}q_{i}\left(t\right)$$

where Φ ∈ ℝ *^n^*^×^*^n^* is the modal matrix that consists of the mode shape vector **φ***_i_* ∈ ℝ*^n^* , and **q**(*t*) = [*q*_1_(*t*),…, *q_n_*(*t*)]*^T^* is a matrix of the corresponding modal coordinates. In particular, in free vibration, that is **f**(*t*) = 0, the single-mode signals can be expressed as monotone exponentially-decaying sinusoids

(4) $$\begin{array}{cc} q_{i}\left(t\right)=u_{i}\exp^{-\varsigma_{i}\omega_{i}t}\text{cos}\left(\omega_{di}t+\theta_{i}\right), & i=1,\ldots ,n \end{array}$$

where *ω_i_* and *ζ_i_* are natural frequencies and damping ratios, respectively; *ω_di_* is the damped natural frequency, $\omega_{di}=\omega_{i}\sqrt{1-\varsigma_{i}^{2}}$; and *u_i_* and *θ_i_* are constants under corresponding initial conditions.

To identify Φ and **q**(*t*) on the sole basis of **x**(*t*), a parametric model must be established by using traditional identification methods. The common method of SSI [2,11] is based on the state space model and uses the theory of subspace identification to identify the parameters of the model structure. However, this method has some drawbacks in terms of model order determination and spurious mode problems, which require the standard stabilization diagram to obtain exact result. Note that Equations (1) and (3) share the same format. The modal coordinate **q**(*t*) is a special case of the general sources **s**(*t*) with time structure, whereas Φ corresponds to the mixing matrix **A**. Furthermore, distinct modal coordinates are uncorrelated and thus automatically satisfy the requirement of sources in BSS. The coordinates are also spectrally monotone in the frequency domain and thus meet the sparsity assumption of SCA . Therefore, Φ and **q**(*t*) can be obtained from the output **x**(*t*) by applying BSS and SCA, both of which provide a straightforward and efficient alternative for output-only modal identification.

## 3. Sparse Component Analysis (SCA)

In this section, we discuss all techniques used in the proposed SCA. First, the sparsity of signals in different transformed domains is illustrated, and the reason for the use of TF domain is explained. Then, SSP detection and K-hyperline clustering are presented to improve the accuracy of the mixing matrix estimation. Finally, the algorithm for sources reconstruction is discussed.

### 3.1. Sparsity in Transformed Domain

A signal is considered sparse in some *ξ*-domains if only a small number of coefficients in the *ξ*-domain significantly differ from zero. The linear transformation that changes the time-domain BSS model to the sparse *ξ*-domain can be represented as follows:

(5) $$\text{x}\left(\xi \right)=As\left(\xi \right)=\sum_{i=1}^{n}{\text{a}}_{i}s_{i}\left(\xi \right)$$

If **s**(*ξ*) is sufficiently sparse and disjoint at some *ξ_k_*, that is, *s_j_*(*ξ_k_*) ≠ 0 and *s_i_*(*ξ_k_*) = 0 for *i* ≠ *j*, then

(6) $$\text{x}\left(\xi_{k}\right)=\sum_{i=1}^{n}{\text{a}}_{i}s_{i}\left(\xi_{k}\right)={\text{a}}_{j}s_{j}\left(\xi_{k}\right)$$

At these points (*i.e.*, SSPs), only one source is active and the mixture vector **x**(*ξ_k_*) is proportional in magnitude to one of the mixing matrix columns. Therefore plotting the magnitude of the mixture vectors in *ξ*-domain as a scatter diagram (e.g., a plot of *x*_1_(*ξ*) versus *x*_2_(*ξ*)) will form clusters and show a clear orientation toward the directions of the column vectors of the mixing matrix **A**. The number of active sources can be counted by recognizing significant directions. The clustering algorithm can be applied to estimate **A**.

A simple example is presented to display the orientation-clustering appearances in different *ξ*-domains. Three sources with distinct frequencies, namely, *s*_1_(*t*) = cos(2*π*·0.2*t*), *s*_2_(*t*) = cos(2*π*·0.6*t*), and *s*_3_(*t*) = cos(2*π* · 1.3*t*), are mixed by the matrix $A=\left[\begin{array}{ccc} 4 & -2 & 1\\ 1 & 2 & -3 \end{array}\right]$.

Two mixtures are generated as

$$\begin{array}{c} x_{1}\left(t\right)=4\cdot s_{1}\left(t\right)-2\cdot s_{2}\left(t\right)+s_{3}\left(t\right)\\ x_{2}\left(t\right)=s_{1}\left(t\right)+2\cdot s_{2}\left(t\right)-3\cdot s_{3}\left(t\right) \end{array}$$

**Figure 1.**
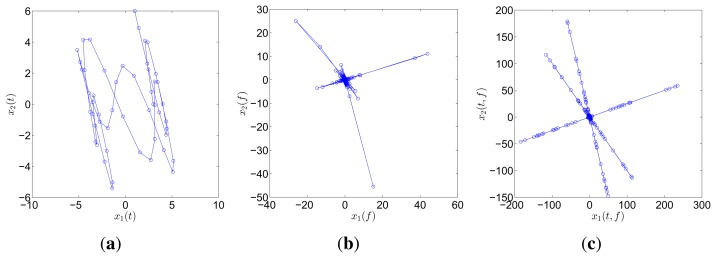
Scatter diagram of two mixtures with three sources in (**a**) time domain; (**b**) frequency domain by DCT; and (c) TF domain by STFT.

The two mixtures are respectively transformed into the frequency and TF domain for comparison. Instead of Fourier transform, discrete cosine transform (DCT) is used in the frequency domain on account of the reason reported in [31]. In the TF domain, short-time Fourier transform (STFT), which has been successfully applied in speech signals, is used because of its simplicity and intuitiveness. Mirror operation and window function with long overlap are applied to solve the problems of edge effect and low accuracy. In this study, STFT is conducted with a Hanning window(length is 256 and overlap is 192). In order to better compare the result between DCT and STFT, the coefficient matrix *x_i_*(*t*, *f*),*i* = 1, 2 obtained by STFT is first reorganized to a vector *x*(*ξ*),*i* =1, 2. The scatter diagrams of *x*_1_(*t*) versus *x*_2_(*t*) or x_1_(*ξ*) versus *x*_2_(*ξ*) are plotted in Figure 1.

The scatter points in the time domain are disordered and reveal minimal information about the mixing matrix. However, the points in the frequency and TF domains form clusters around three significant directions, which correspond to the three columns of the mixing matrix **A**. Furthermore, the STFT result is superior to that of the DCT because only a small fraction of points remarkably reveal the directions in the DCT scatter diagram. DCT performance is thus sensitive to noise and outliers. In this study, we choose to perform SCA in the TF domain by using STFT.

### 3.2. Mixing Matrix Estimation

Estimation of the mixing matrix is important in SCA. Estimation accuracy can be improved in two ways: SSP detection and K-hyperline clustering.

#### 3.2.1. SSP Detection

The SSP is the point where only one source is active, and the direction of the magnitude of the mixture vectors is proportional to that of the columns of the mixing matrix. SSP detection can thus reduce the effect of noise and outliers as well as improve clustering performance.

Equation (1) can be expressed in the TF domain using STFT as

(7) $$\begin{array}{ll} X\left(t,k\right) & =\text{AS}\left(t,k\right)\\ & =\overset{n}{\underset{i=1}{\text{∑}}}a_{i}S_{i}\left(t,k\right) \end{array}$$

where **X**(*t*, *k*) = [*X*_1_(*t*, *k*),…, *X_m_*(*t*, *k*)]*^T^* and **S**(*t*, *k*) = [*S*_1_(*t*, *k*),…, *S_n_*(*t*, *k*)]*^T^* represent the STFT coefficients of the mixtures and sources, respectively, in the *k*th frequency bin at time frame *t*; **a***_i_* = [*a*_1*i*_, … , *a_mi_*]*^T^* is the ith column of the mixing matrix **A**. We assume a case with two sources and two sensors. If the (*t*_1_, *k*_1_) in the TF domain is an SSP, that is, *s*_1_(*t*_1_, *k*_1_) ≠ 0 and *s*_2_(*t*_1_, *k*_1_) = 0, then the following can be obtained from Equation (7):

(8) $$X\left(t_{1},k_{1}\right)=a_{1}S_{1}\left(t_{1},k_{1}\right)$$

The real and imaginary parts can be equated through plural unfolding as

(9) Re{X(t1,k1)}=a1Re{S1(t1,k1)}

(10) Im{X(t1,k1)}=a1Im{S1(t1,k1)}

In this case, the absolute directions of the real and imaginary parts are the same as that of **a**_1_. This condition is derived from the fact that only one source is active at an SSP. The real and imaginary parts of a non-SSP (*t*_2_, *k*_2_) at which both sources are active can be written as

(11) Re{X(t2,k2)}=a1Re{S1(t2,k2)}+a2Re{S2(t2,k2)}

(12) Im{X(t2,k2)}=a1Im{S1(t2,k2)}+a2Im{S2(t2,k2)}

If the absolute directions of the real and imaginary parts need to be the same, then the following condition must be satisfied:

(13) Re{S1(t2,k2)}Im{S1(t2,k2)}=Re{S2(t2,k2)}Im{S2(t2,k2)}

When extending to the general case of multiple mixtures and multiple sources, the condition that the absolute directions of Re{**X**(*t*, *k*)} and Im{**X**(*t*, *k*)} at any point (*t*, *k*) are the same requires that the ratios between the real and imaginary parts of the STFT coefficients of all source signals at those points must also be the same. However, the probability of satisfying this requirement is low and decreases as the number of sources increases [28]. Therefore, we can conclude that a point is an SSP if the absolute directions of Re{**X**(*t*, *k*)} and Im{**X**(*t*, *k*)} at a point (*t*, *k*) are the same; otherwise, the point is a multiple-source-point. However, the points that accurately satisfy this condition are limited. Therefore, [28] developed an algorithm that relaxes this condition for SSP detection. In the TF plane, the points where the difference between the absolute directions of Re{**X**(*t*, *k*)} and Im{**X**(*t*, *k*)} is less than Δ*θ* are taken as SSPs. This condition can be expressed as

(14) |Re{X(t,k)}TIm{X(t,k)}‖Re{X(t,k)}‖⋅‖Im{X(t,k)}‖|>cos(Δθ)

where | · | represents the absolute value, and $\left\|y\right\|=\sqrt{y^{T}y}$. The SSPs that satisfy Equation (14) can be preserved for the estimation of the mixing matrix.

**Figure 2.**
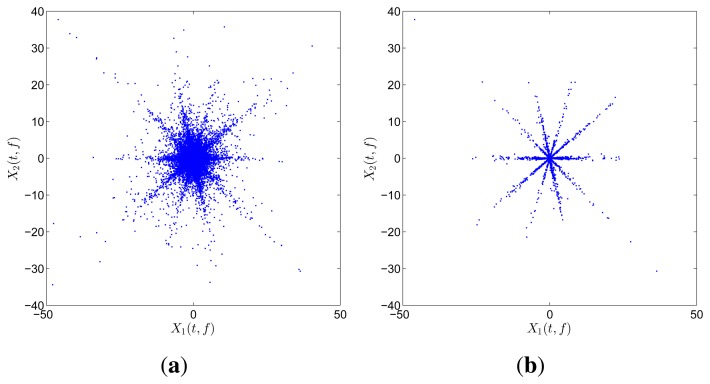
Scatter diagram of an example of speech utterances with five sources and two mixtures: (**a**) all STFT coefficients; (**b**) the detected SSPs.

Figure 2 illustrates an example of speech utterances with five sources and two mixtures. All STFT coefficients are shown in Figure 2a, whereas the SSPs detected by using Equation (14) are shown in Figure 2b. The directions in Figure 2b are much clearer than those before SSP detection, and the scale of points for clustering is also reduced. Therefore, SSP detection makes SCA simple, effective, and robust to noise and outliers.

#### 3.2.2. K-Hyperline Clustering

After SSP detection, estimating the mixing matrix **A** can be cast into a hyperline clustering problem. Several standard clustering methods have been used to extract directions; examples of these methods include K-means, fuzzy-C clustering [32] and the linear geometric ICA-based method [33]. However, these methods require the sources to be sufficiently sparse, and their performance in practice may be affected by many factors such as noise, outliers, and insufficient sparseness of sources, as mentioned in [16].

Considering these problems, [34,35] proposed and studied K-hyperline clustering method. In contrast to the K-means clustering method, K-hyperline allows each data sample to have a coefficient value that corresponds to the cluster centroid, and the cluster centroids are normalized to unit *ℓ*_2_–norm. Clustering can be rapidly realized by solving an optimization problem using singular value decomposition or an iterative linear scheme. Interestingly, this method is robust to the outliers and performs well even in noisy cases. Further discussion on this clustering method is not presented in this paper due to space limitation.

### 3.3. Reconstruction of Sources

Once the mixing matrix is estimated and denoted as **Ã**, the sources can be directly recovered by solving **s̃**(*t*) = **Ã**^−1^x(*t*) for *m* = *n* or by using the least squares method for *m* > *n*. However, for the case of UBSS(*i.e.*, *m* < *n*), an infinite amount of feasible solutions may be obtained in accordance with the theory of linear equations. From these solutions, we want to find the one that has the least error with our real sources. Given the sparsity of the sources in *ξ*-domain, the solution is required to have the fewest non-zero elements, which can be formulated as: (*P*_0_) : *s̃*(*ξ*) = argmin ‖*s*(*ξ*)‖_0_, where ‖x‖_0_ denotes the 0-norm of x. However, (*P*_0_) is subject to its non-convexity and high-discreteness and is therefore difficult to executed in practice. Replacing the subscript 0 with 1, we obtain the problem (*P*_1_) : *s̃*(*ξ*) = argmin‖*s*(*ξ*)‖_1_, which is also called the basis pursuit(BP) problem. [36] proved that (*P*_1_) is a good approximation to (*P*_0_). (*P*_1_) can be realized by a well-defined convex optimization problem, whose solution can be guaranteed as global optimum and can be solved efficiently by using standard linear programming techniques. For the noiseless case, BP is a effective solution to recover the sparse sources. However, BP can not find a good solution in the presence of noise. Thus, we use BPDN technique to recover the sources in this manuscript. The optimization problem of BPDN is formulated as follows:

(15) $$\begin{array}{lll} \tilde{s}\left(\xi \right)=\arg\min{\left\|s\left(\xi \right)\right\|}_{1} & \text{subject to} & {\left\|\text{x}\left(\xi \right)-\tilde{A}\text{s}\left(\xi \right)\right\|}_{2}<\sigma \end{array}$$

The case *σ* = 0 corresponds to a solution of BP. The MATLAB solver SPGL1 is used to recover the sources from the time-frequency coefficients. For the noiseless case in Sections 4.1 and 4.2, the BP method is used, and the BPDN method is used in the experimental part and the noise case of the simulation part. Additional description of the *ℓ*_1_-minimization technique can be found in [36-40].

When the source signals **s̃**(*ξ*) in *ξ*-domain are recovered, the time-domain sources **s̃**(*t*) in Equation (1) can be calculated by the corresponding inverse transform as

(16) $$\tilde{\text{s}}\left(t\right)=T^{-1}\left(\tilde{\text{s}}\left(\xi \right)\right)$$

Then the modal parameters can be extracted from the recovered sources by using the SDOF parameter fitting method or logarithmic decrement method.

## 4. Numerical Simulations

**Figure 3.**
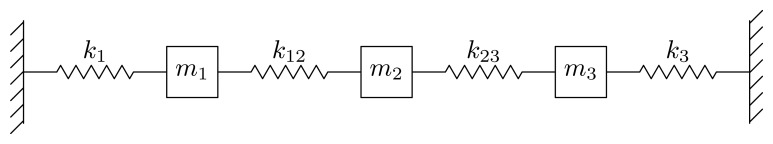
Three-dof linear model.

A 3-dof mass-spring-damper system (Figure 3) is considered in this section to validate the effect of the proposed TF-domain SCA method. Different system parameters are selected for various situations, such as well-separated modes, highly-damped modes, and closely-spaced modes. In each situation, determined and underdetermined identification are both considered. The robustness to noise is studied in Section 4.3.

The proposed algorithm is performed under the following settings. The simulation outputs are first transformed into the TF plane using STFT with a Hanning window length of 512 and an overlap of 384. Then, the SSPs in the TF plane are found by the value Δ*θ* of 10°. a time-frequency trade-off occurs when using STFT. The width of the windowing function relates to how the signal is represented-it determines whether there is good frequency resolution or good time resolution. a wide window gives better frequency resolution but poor time resolution. a narrower window gives good time resolution but poor resolution. The methods for obtaining high resolution STFT have been studied in [41,42]. [41] proposed a method to separate nonstationary and stationary signals overlapping in time-frequency domain; time and frequency varying windows and overlapped STFT was used to improve time and frequency resolution. [42] developed a high-resolution time-frequency representation to achieve robust estimation of multiple frequency hopping signals based on sparse Bayesian method. The source signals in OMA are exponentially-decaying sinusoids. Thus, the time resolution is not so important and we can select a wide window. Meanwhile, the overlap is set at 75% of the window length to obtain adequate single source points. In practice, the minimal natural frequency is another factor that should be considered in deciding the window length. As the model in Section 4.1 shows, the minimal natural frequency is 0.0895 (the period is 112 points at the sampling frequency 10 Hz); thus, the window length should contain at least several periods. On the basis of the reasons above, the window length and overlap are set as the ones above. Δ*θ* is set to 10° to make sure that we can obtain adequate clean SSPs. The mixing matrix is obtained through K-hyperline clustering. With the estimated mixing matrix, the modal responses are directly recovered by solving Equation (1) in the time domain for the determined case. For the underdetermined case, the frequency-domain modal responses are first recovered by BPDN. Then the overlap add (OLA) method is used to execute inverse STFT to get the time sequence, details of OLA method can be found in [43]. After the inverse STFT is carried out, the time-domain modal responses are obtained. Finally, the natural frequency and damping ratio of each estimated modal response can be calculated by using either the simple SDOF parameter fitting or logarithmic decrement method.

The modal assurance criterion (MAC) is used to determine the errors of the estimated mode shape as

(17) $$\text{MAC}\left({\tilde{\varphi }}_{i},\varphi_{i}\right)=\frac{{\left({\tilde{\varphi }}_{i}^{T}\cdot \varphi_{i}\right)}^{2}}{\left({\tilde{\varphi }}_{i}^{T}\cdot {\tilde{\varphi }}_{i}\right)\left({\tilde{\varphi }}_{i}^{T}\cdot \varphi_{i}\right)}$$

where **φ̃**_i_ and **φ̃**_i_ are the *i*th estimated and theoretical mode vectors, respectively. The MAC value ranges from 0 to 1, in which 0 means no correlation and 1 indicates complete correlation. For the underdetermined case, the estimated mode vectors have lower dimensions than the theoretical ones. The corresponding rows of the theoretical mode vectors are selected to constitute the partial mode shapes. Thus the MAC indicates the estimating accuracy of the partial mode shapes.

### 4.1. Well-Separated Modes

First, the case of well-separated modes is considered. The system parameters, identical to those in [31], are set as follows:

$$M=\left[\begin{array}{ccc} 2 & 0 & 0\\ 0 & 1 & 0\\ 0 & 0 & 3 \end{array}\right],K=\left[\begin{array}{ccc} 1 & 0 & 0\\ 0 & 1 & 0\\ 0 & 0 & 1 \end{array}\right]$$

The damping matrix is determined by the mass matrix as **C** = *α***M**, where *α* = 0.08, 0.13 to account for different damping levels. The free response is adopted, and the initial states are x(0) = [0 0 0]^T^ and x¨(0) = [0 1 0]^T^.

#### 4.1.1. Determined Modal Identification

**Figure 4.**
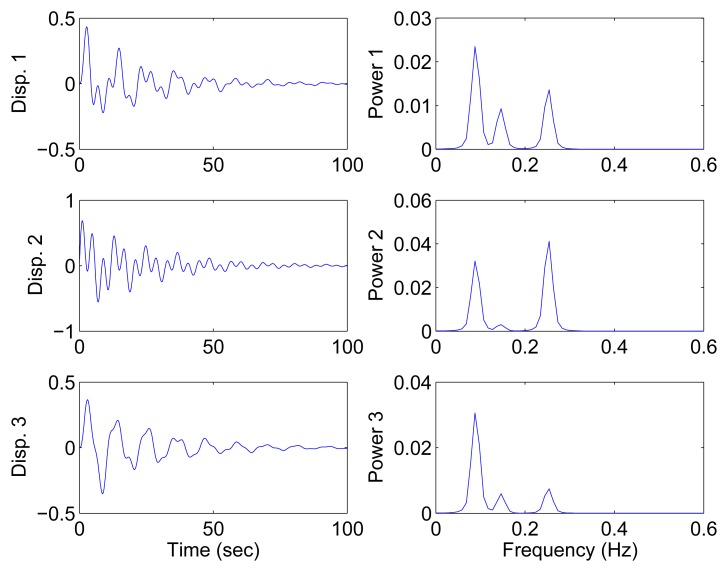
Free-vibration system responses and their power spectral density in the case of well-separated modes, *α* = 0.08

**Figure 5.**
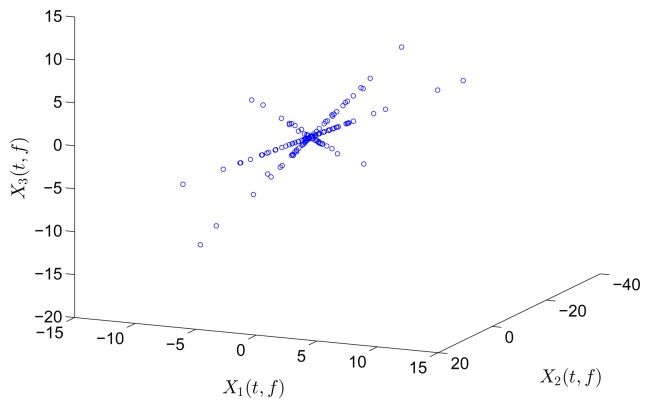
Scatter diagram of the identified SSPs using all three sensors in the case of well-separated modes, *α* = 0.08.

**Figure 6.**
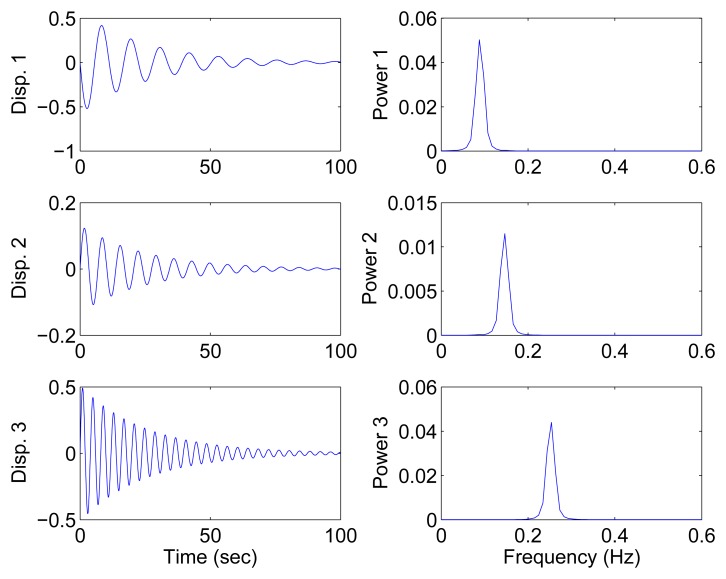
Recovered modal responses using all three sensors in the case of well-separated modes, *α* = 0.08.

In this section, all sensor measurements are used for identification. The responses of the free vibration are shown in Figure 4, and the scatter diagram of the identified SSPs is shown in Figure 5, from which we can see that the SSPs form three clear directions. The mixing matrix, that is, the matrix of mode shape vectors, can then be accurately estimated through K-hyperline clustering. The performance can be indicated by the high MAC values presented in Table 1. The recovered time-domain modal responses, as shown in Figure 6, have waveforms that are similar to those of monotone exponentially decaying sinusoids. The frequencies and damping ratios are estimated from these modal responses, as shown in Table 1. Compared with the theoretical values, the proposed algorithm obtains excellent results regardless of damping levels.

#### 4.1.2. Underdetermined Modal Identification

**Figure 7.**
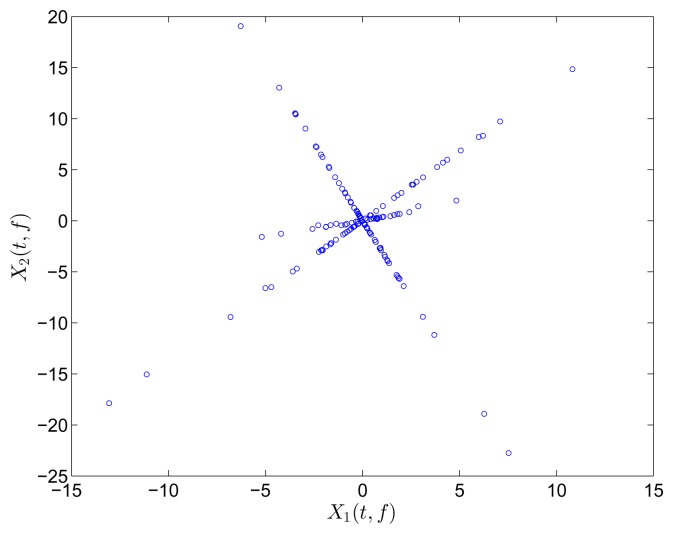
Scatter diagram of the identified SSPs using the first two sensors in the case of well-separated modes, *α* = 0.08.

**Figure 8.**
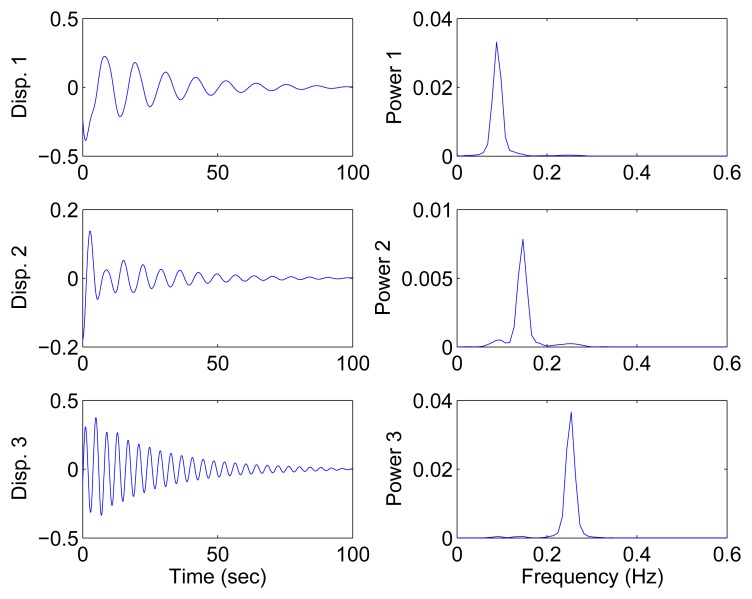
Recovered modal responses using the first two sensors in the case of well-separated modes, *α* = 0.08.

In the underdetermined case, the first two measurements are utilized. Figure 7 shows the 2D scatter of the SSPs. Furthermore, three clear directions represent the mode shape vectors. The *ℓ*_1_-optimization algorithm is used to recover the time-domain modal responses, and the results of the case where *α* = 0.08 are shown in Figure 8. The high MAC values and the accurately identified parameters are presented in Table 1. These results reveal that the proposed algorithm is also suitable for underdetermined modal identification with limited sensors.

The TF-domain SCA in this study achieves a better performance compared with the frquency-domain SCA in [31]. On the one hand, more points remarkably reveal the orientation, which make the directions more obvious and recognizable. This feature is helpful in enhancing estimation accuracy of the mixing matrix. On the other hand, the system parameters and mode shapes are identified with high accuracy.

### 4.2. Closely-Spaced Modes

Closely-spaced modes are studied in this section to further evaluate the capacity of the proposed SCA. The model is set up with the following parameters:

$$M=\left[\begin{array}{ccc} 1 & 0 & 0\\ 0 & 2 & 0\\ 0 & 0 & 1 \end{array}\right],K=\left[\begin{array}{ccc} 5 & -1 & 0\\ -1 & 4 & -3\\ 0 & -3 & 3.5 \end{array}\right],C=\left[\begin{array}{ccc} 0.0894 & -0.0084 & 0.0003\\ -0.0084 & 0.1301 & -0.0244\\ 0.0003 & -0.0244 & 0.0772 \end{array}\right]$$

**Figure 9.**
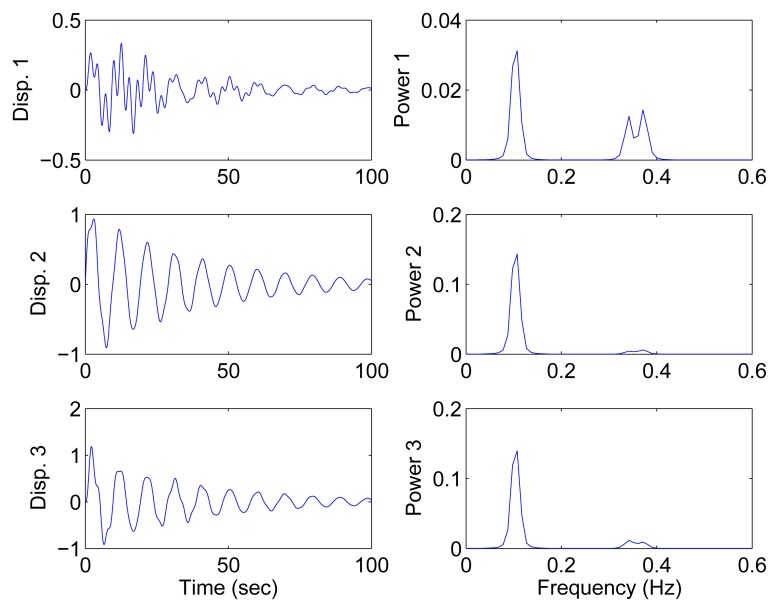
Free-vibration system responses and their power spectral density in the case of closely-spaced modes.

**Figure 10.**
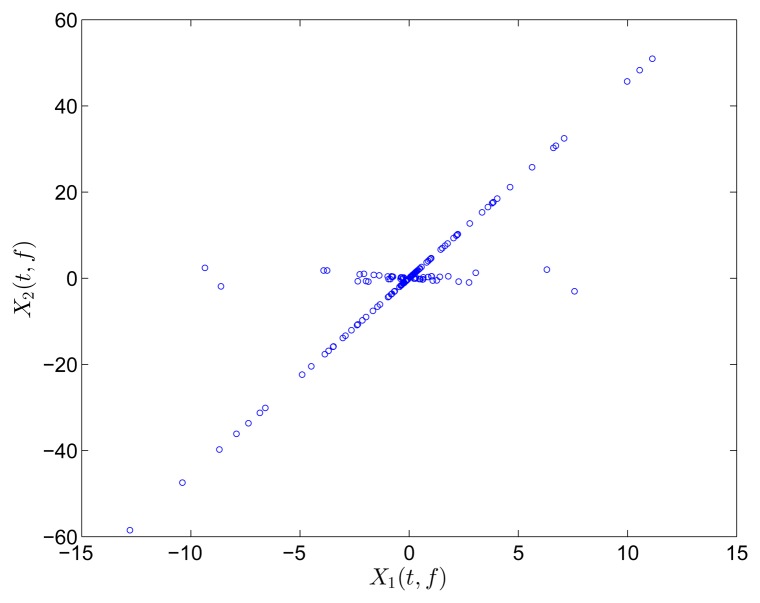
Scatter diagram of the identified SSPs using two sensors in the case of closely-separated modes.

**Figure 11.**
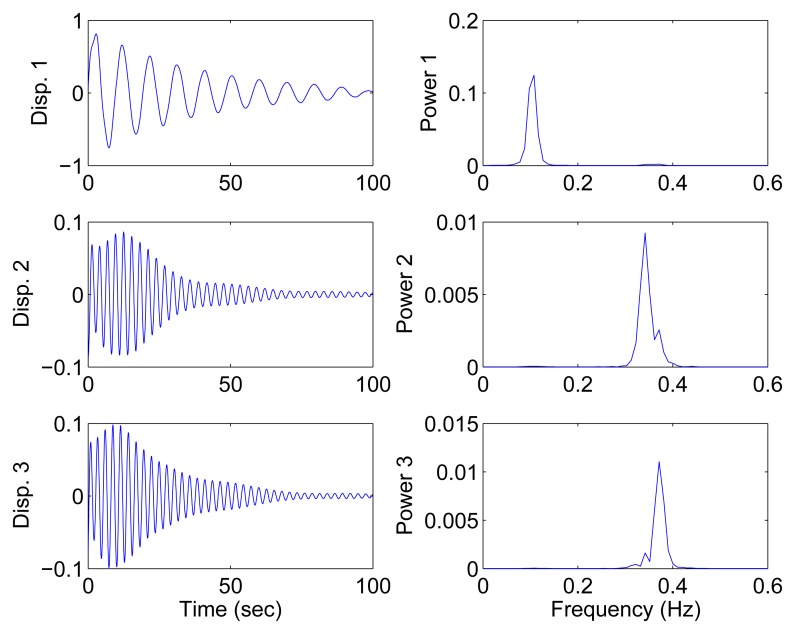
Recovered modal responses using two sensors in the case of closely-separated modes.

Figure 9 shows the free vibration of the model under the same initial conditions as those in Section 4.1. As indicated in the spectrum, the second and third modes cannot be clearly separated. However, the scatter plot of the identified SSPs in Figure 10 reveals three significant directions. Although the angle between the two of them is slightly small, high MAC values can be obtained as presented in Table 2, where the estimated natural frequencies and damping ratios in the determined and underdetermined cases are also displayed. The identified parameters match the theoretical ones well. Figure 11 shows the recovered modal responses in the underdetermined case.

Similar to the frequency-domain SCA, the TF-domain SCA is effective for closely-spaced modes.

### 4.3. Robustness to Noise

**Figure 12.**
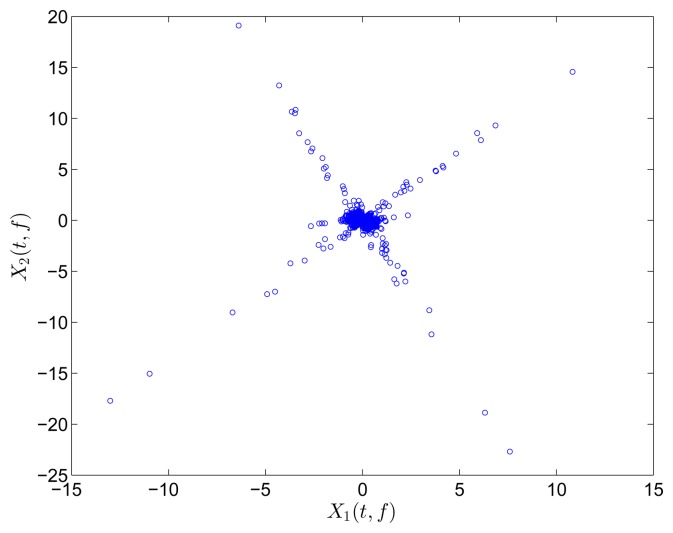
Scatter diagram of the identified SSPs using two sensors with 10% RMS noise.

**Figure 13.**
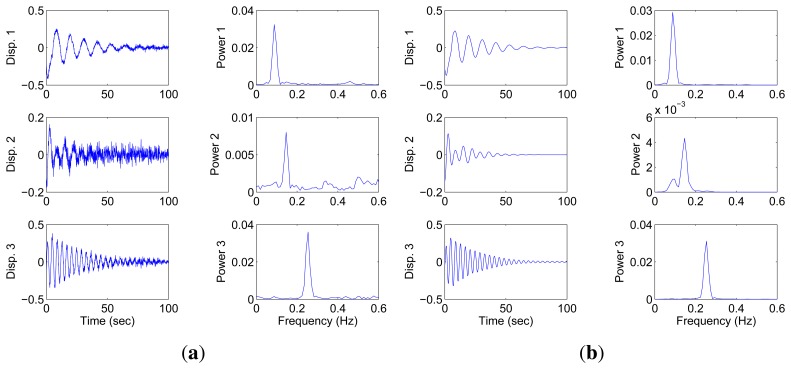
Under the condition of two sensors, 10% RMS noise, the modal responses recovered by different methods: (**a**) BP; (**b**) BPDN.

To investigate the robustness to noise, the simulation outputs are corrupted by Gaussian white noise, the root mean square(RMS) amplitude of which is set to a certain percentage of the signal RMS value. The model is the same as that used in Section 4.1. The identified SSPs and the recovered modal responses under 10% noise are shown in Figures 12 and 13. Although the addition of noise results in a confusing scatter, many points are still distributed around the direction lines, and their average-value are used to reduce the effect of noise. BP and BPDN are used to recover the sources. Comparison shows that BPDN performs better than BP in the presence of noise. To evaluate the performance of the proposed algorithm with increasing noise percentage, 5%, 10%, 15% and 20% RMS noise are added to the original source. Fifty groups of sources with different white noise under each noise level are processed with the proposed algorithm because of the non-deterministic characteristic of the noise. The mean values of the MAC of the successful identifications are shown in Table 3. The identification is considered successful when the MAC of each identified mode is greater than 0.95. The high MAC values shown in Table 3 indicate that the proposed algorithm can also achieve good results in the presence of noise. The proposed algorithm may fail when the noise level increases to a certain extent (20% for this example).

**Figure 14.**
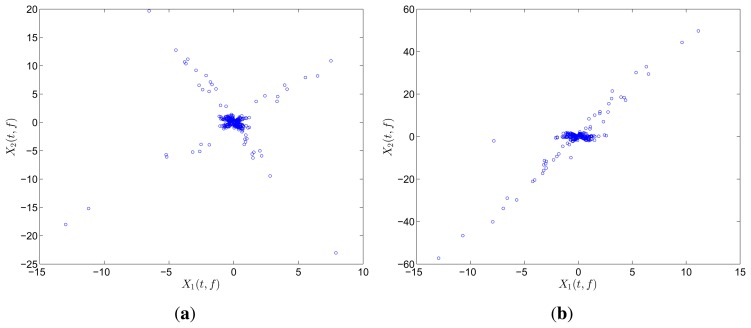
Two conditions when the proposed method fails because of noise.

The scatter diagram of one failed case under 20% noise leve is shown in Figure 14a. The valuable single source points that correspond to the shortest hyperline are flooded in the noise, and the direction vector cannot be found in this case. There is another condition in which the method may fail due to noise exists. We have examined the performance for the close-separated modes in Section 4.2. The scatter plot of the mixture when the sources are polluted by noise (10%) is shown in Figure 14b. This figure shows that the single source points deviate from their original position(due to noise) and become mixed. As a result, two hyperlines are identified as one, and the other direction vector may be arbitrary. The MAC values [0.9972 0.4851 0.9995] validate this finding. If not in these two conditions, the proposed algorithm can behave well enough.

## 5. Experimental Verification

### 5.1. Experimental Setup

A steel cantilever beam that measures 0.31 *m* × 0.0012 *m* × 0.0004 *m* is used to validate the effectiveness of the proposed modal identification algorithm. The steel beam is made of carbon tool steel whose Young's modulus and density are 2.06 × 10^11^ *N* ·*m*^−2^ and 7.85 × 10^3^ *kg* · *m*^−3^, respectively. One end of the steel beam is fixed on a table, and the length from the fulcrum to the end is 0.28 *m*.

**Figure 15.**
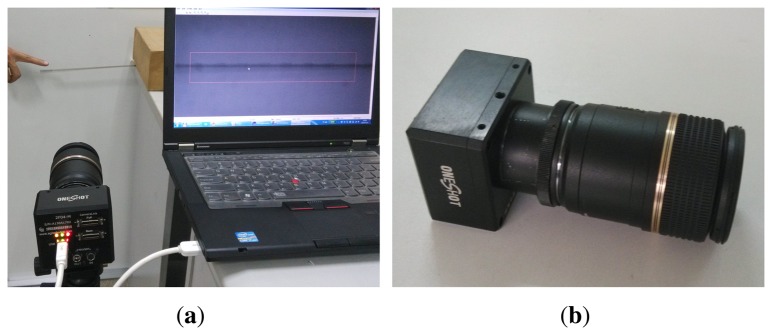
Experimental Setup: (**a**) experimental setup; (**b**) high-speed camera head.

**Figure 16.**
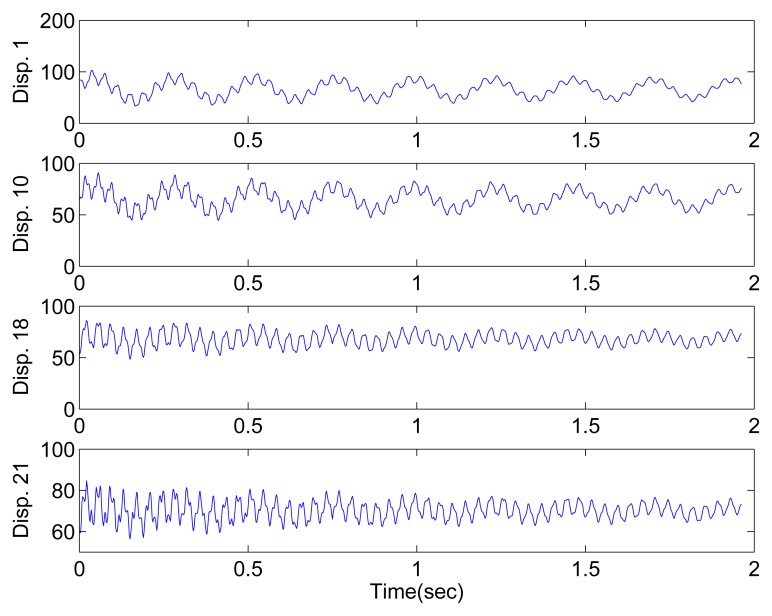
System responses of the steel beam extracted by image processing.

When excitation is provided by human finger tapping, the steel beam begins to vibrate. To capture the displacement responses, a high-speed camera is used instead of traditional accelerometer. Figure 15a shows the experimental setup, and Figure 15b shows the high-speed camera head. Twenty-one locations with a spacing of approximately 0.01 *m* are marked for displacement measurement. Gray 8-bit images can be transferred at 500 fps (in a 150 × 1200 pixel area, one pixel corresponds to 0.0002 *m*) by using the device. The camera head is connected to a PC. After image acquisition, the displacement responses at marked locations are extracted by image processing. The displacement responses extracted from some of the locations are shown in Figure 16.

### 5.2. Cantilever Beam Theory

**Figure 17.**
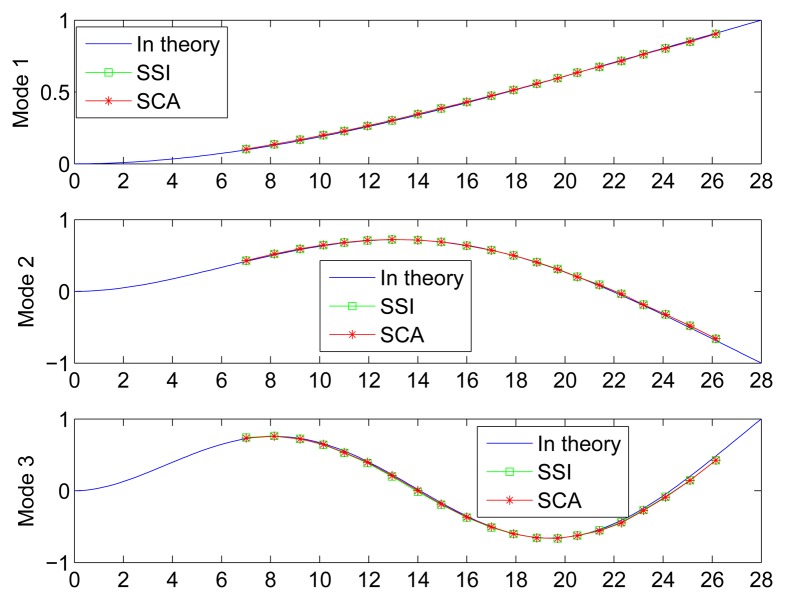
Theoretical and identified mode shapes.

The vibration of the cantilever beam satisfies Euler-Bernoulli beam theory, by which the governing differential equation without damping is formulated as

(18) $$\frac{\partial^{4}y}{\partial x^{4}}+a\frac{\partial^{2}y}{\partial t^{2}}=0$$

where $a=\sqrt{EJ/\rho A}$, *E* is Young's modulus, *J* is the second moment area of the beam cross-section, *ρ* is the material density, and *a* is the cross-sectional area of the beam. Under the boundary conditions of the cantilever beam, the natural frequency function and corresponding mode shape function can be calculated as

(19) $$\omega_{i}=\frac{{\left(\beta_{i}l\right)}^{2}}{l^{2}}a$$

(20) $$Y_{i}\left(x\right)=\frac{\text{cos}\left(\beta_{i}l\right)+ch\left(\beta_{i}l\right)}{\text{sin}\left(\beta_{i}l\right)+sh\left(\beta_{i}l\right)}\left[\text{sin}\left(\beta_{i}x\right)-sh\left(\beta_{i}x\right)\right]+ch\left(\beta_{i}x\right)-\text{cos}\left(\beta_{i}x\right)$$

where *β_i_l* is determined by *cos*(*βl*)*ch*(*βl*) = − 1, and *l* is the length of the cantilever beam. The natural frequencies of the former three orders based on the substitution of the actual parameters of the steel beam are shown in Table 4. The corresponding mode shapes are shown in Figure 17.

### 5.3. Results Analysis

**Figure 18.**
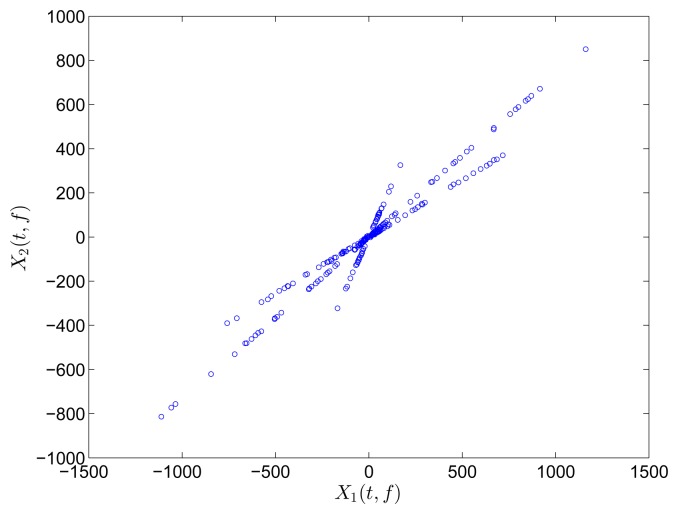
Scatter diagram of the identified SSPs using only two measurements.

**Figure 19.**
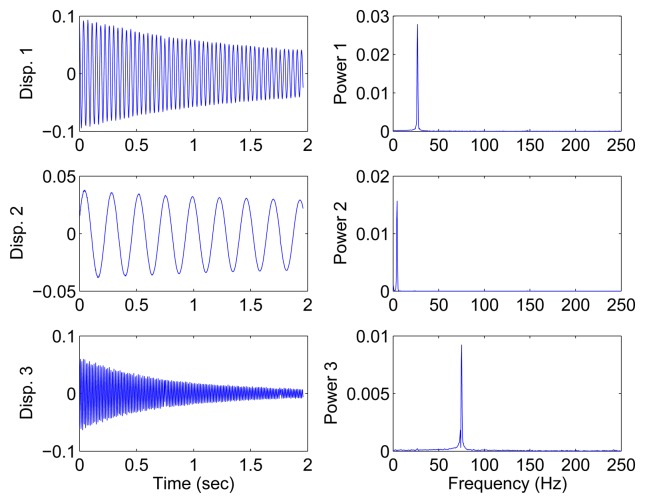
Modal responses recovered by the proposed SCA using all of the 21 measurements.

**Figure 20.**
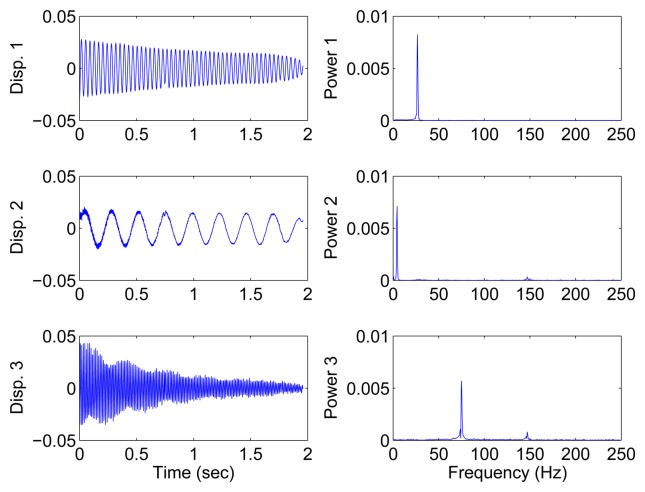
The modal responses recovered by the proposed SCA using only two measurements.

To accomplish the modal analysis of the steel cantilever beam, the proposed SCA algorithm is applied to the displacement responses. Figure 18 depicts the scatter diagram of the identified SSPs using two of the measurements, which reveals three significant directions. This result indicates that three modes of the cantilever beam are excited. The modal responses recovered in the determined case using all the displacement responses as well as that using only two measurements (1st and 10th markers) with the proposed SCA are shown in Figures 19 and 20. The recovered responses are similar to the monotone exponentially decaying sinusoids.

As a common and effective method for modal identification, SSI is also used in this study for comparison with the proposed SCA. To avoid the drawbacks of the SSI, all displacement responses are used as the input in subspace identification. The detailed results of the corresponding natural frequencies and damping ratios are listed in Table 4. The identified mode shapes by both SSI and the proposed SCA in the determined case are shown in Figure 17 for contrast with the theoretical ones.

In Table 4, the identified natural frequencies and damping ratios obtained by the proposed SCA in both the determined and underdetermined cases agree very well with those obtained by SSI. The identified frequencies also match the theoretical results. In addition, the identified mode shapes by the two methods show a high correlation with the theoretical waveforms in Figure 17, as also indicated by the MAC values. a comparison between the underdetermined case and the determined one shows that the former uses fewer measurements but attains a similarly good performance.

This experiment successfully validates the effectiveness of the proposed SCA for OMA in practice. Especially for the underdetermined case, similar performance can be obtained despite the use of limited measurements, whereas the SSI method needs more measurements to ensure good results. The proposed method provides a new way for the output-only modal identification.

## 6. Conclusions

This paper presents a new SCA method for OMA. Compared with existing methods, the proposed method explores the sparse representations in the TF domain to perform output-only modal identification. Some clusters are formed when the mixed signals are transformed into the TF plane. SSP detection and K-hyperline clustering are combined to improve the estimation accuracy of the mixing matrix. The SSP is the point in which only one source is active, and the direction of the magnitude of the mixture vectors are proportional to those of the columns of the mixing matrix. Therefore, SSP detection can reduce the effect of noise and outliers as well as improve the clustering performance. After SSP detection, the estimation of the mixing matrix is cast into a hyperline clustering problem. Therefore, K-hyperline clustering is introduced to fulfill the clustering task. After the mixing matrix is estimated, the theory of linear equations for determined BSS or the *ℓ*_1_-minimization technique for UBSS is used to recover the modal responses, from which the modal information (natural frequency, damping ratio, and mode shapes) can be extracted. The numerical simulations and experimental results demonstrate the effectiveness of the proposed SCA method in various cases. a comparison of the proposed method with SSI reveals that the two methods show equal performance in the experimental section. Further investigation is required to explore the applications of the proposed SCA in complex modes cases, which are not covered in this study.

## Figures and Tables

**Table 1.** Modal parameters identified by the proposed SCA for the simulation model in the case of well-separated modes.

***α***	**Mode**	**Frequency (Hz)**			**Damping Ratio(%)**				**MAC**	
		
		**Theoretical**	**Determined**	**Under-**	**Theoretical**	**Determined**	**Under-**		**Determined**	**Under-**
0.08	1	0.0895	0.0892	0.0886	7.11	7.11	7.50	1.0000		1.0000
	2	0.1458	0.1456	0.1488	4.37	4.37	4.87	0.9998		0.9998
	3	0.2522	0.2521	0.2522	2.52	2.52	2.55	1.0000		1.0000
0.13	1	0.0895	0.0889	0.0876	11.55	11.55	11.79	1.0000		1.0000
	2	0.1458	0.1454	0.1517	7.10	7.09	7.57	1.0000		0.9996
	3	0.2522	0.2520	0.2521	4.10	4.10	4.13	1.0000		1.0000

**Table 2.** Modal parameters identified by the proposed SCA to the simulation model in the case of closely-spaced modes.

**Mode**	**Frequency (Hz)**			**Damping Ratio(%)**			**MAC**	
		
	**Theoretical**	**Determined**	**Under-**	**Theoretical**	**Determined**	**Under-**	**Determined**	**Under-**
1	0.1039	0.1038	0.1038	4.00	4.00	4.59	1.0000	1.0000
2	0.3425	0.3424	0.3435	2.00	2.00	1.77	0.9996	0.9963
3	0.3713	0.3712	0.3718	2.00	2.00	1.71	1.0000	0.9983

**Table 3.** Accuracy of mixing matrix estimation(MAC) and modal parameters identified by the proposed SCA to the simulation model under different noise levels.

**Noise Percentage (%)**	**Mode 1**	**Mode 2**	**Mode 3**	**Successful Identifications**
5	1	0.9999	0.9999	50/50
10	0.9998	0.9998	0.9998	50/50
15	0.9995	0.9995	0.9995	47/50
20	0.9980	0.9778	0.9980	18/50

**Table 4.** Modal parameters identified by the SSI and the proposed SCA for the experimental steel beam.

**Mode**	**Frequency (Hz)**				**Damping Ratio(%)**			**MAC**	
		
	**Theoretical**	**SSI**	**Determined**	**Under-**	**SSI**	**Determined**	**Under-**	**Determined**	**Under-**
1	4.22	4.24	4.33	4.33	1.48	1.48	1.46	1.0000	1.0000
2	26.46	26.65	26.76	26.76	0.36	0.36	0.36	1.0000	1.0000
3	74.09	74.83	74.92	74.92	0.26	0.26	0.26	0.9998	0.9998
